# Establishment and evaluation of prediction model for multiple disease classification based on gut microbial data

**DOI:** 10.1038/s41598-019-46249-x

**Published:** 2019-07-15

**Authors:** Sohyun Bang, DongAhn Yoo, Soo-Jin Kim, Soyun Jhang, Seoae Cho, Heebal Kim

**Affiliations:** 1 Interdisciplinary Program in Bioinformatics, Seoul National University, Seoul, 151-742 Republic of Korea; 2 C&K genomics, Seoul National University Research Park, Seoul, 151-919 Republic of Korea; 3 Department of Agricultural Biotechnology and Research Institute of Agriculture and Life Sciences, Seoul National University, Seoul, Republic of Korea

**Keywords:** Machine learning, Metagenomics

## Abstract

Diseases prediction has been performed by machine learning approaches with various biological data. One of the representative data is the gut microbial community, which interacts with the host’s immune system. The abundance of a few microorganisms has been used as markers to predict diverse diseases. In this study, we hypothesized that multi-classification using machine learning approach could distinguish the gut microbiome from following six diseases: multiple sclerosis, juvenile idiopathic arthritis, myalgic encephalomyelitis/chronic fatigue syndrome, acquired immune deficiency syndrome, stroke and colorectal cancer. We used the abundance of microorganisms at five taxonomy levels as features in 696 samples collected from different studies to establish the best prediction model. We built classification models based on four multi-class classifiers and two feature selection methods including a forward selection and a backward elimination. As a result, we found that the performance of classification is improved as we use the lower taxonomy levels of features; the highest performance was observed at the genus level. Among four classifiers, LogitBoost–based prediction model outperformed other classifiers. Also, we suggested the optimal feature subsets at the genus-level obtained by backward elimination. We believe the selected feature subsets could be used as markers to distinguish various diseases simultaneously. The finding in this study suggests the potential use of selected features for the diagnosis of several diseases.

## Introduction

Machine learning technology has been applied in various fields and has become a useful strategy in the field of biotechnology, especially for predicting diseases and supporting medical diagnosis^1–3^. In order to predict diseases, biological data including gene expression, genotype, and methylation level can be employed^4,5^. Moreover, the realms of biological data have been extended to include the microbial communities due to their association with the host’s immune system^6^. Microbial communities facilitate the development and function of the immune cells at both the mucosal and nonmucosal sites^7^. Their regulation of the immune system is involved in various diseases^8^. Such association has been identified in diseases like multiple sclerosis (MS), juvenile idiopathic arthritis (JIA), myalgic encephalomyelitis/chronic fatigue syndrome (ME/CFS), stroke, acquired immune deficiency syndrome (AIDS), and colorectal cancer (CRC)^9–14^.

Some of the well-known researches have attempted to establish a disease-prediction model based on the gut microbiome data from healthy individuals and patients, and have discovered that gut microbiome data can be applied to predict specific diseases^12^. Patients with irritable bowel syndrome and healthy individuals were classified using Random forest algorithm^15^. Other diseases such as liver cirrhosis, colorectal cancer, inflammatory bowel diseases, obesity, and type 2 diabetes were distinguished with a healthy status using machine learning approaches^16^. Most of these studies have focused mainly on diagnosing only one disease, and so far, there have been few attempts to predict multiple diseases at once.

The potential of multi-classification using microbiome data is being shown in recent studies^17,18^. In the case of classifying various body parts, a previous study performed multi-classification based on KNN and probabilistic neural networks^18^. In another study, multi-classification of three different diseases was demonstrated using selected metagenomic biomarkers^19^. Similarly, in our study, we hypothesized that various diseases could be classified using gut microbiome data from 16 S rRNA sequencing. To investigate the possibility of classification on various diseases based on the microbial community, we collected a total of 1,079 metagenome data from healthy individuals and patients with following diseases in six studies: MS, JIA, ME/CFS, AIDS, CRC, and Stroke. To combine data, we preprocessed data using normalization and statistical method. We classified six diseases listed above using the abundance of microorganisms at the phylum, class, order, family, and genus levels as features. We built classification models based on the multi-class classifiers such as LogitBoost, support vector machine (SVM), K nearest neighbor (KNN) and logistic model tree (LMT). Moreover, we constructed a feature subset using two feature selection methods. We compared the performance of classification in three factors: 1) taxonomy levels of features, 2) four classifiers and 3) feature selection methods.

## Results

### Preprocessing of data to reduce biases from meta-analysis

**Figure 1:**
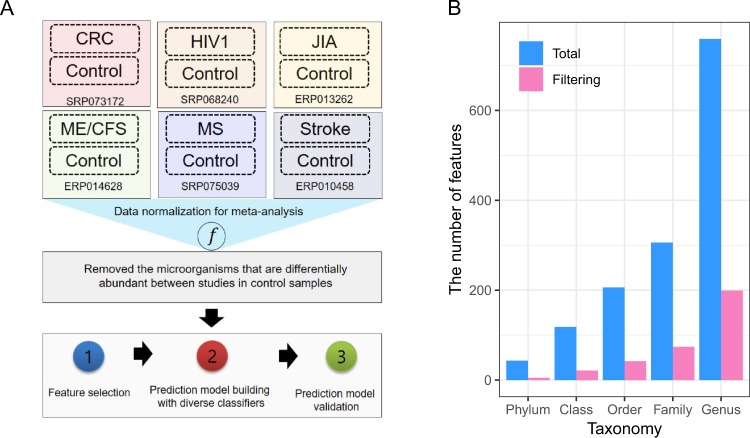
Experimental design and data processing for meta-analysis. (**A**) A diagram representing a whole experimental design for this research. This research consists of two major steps for analysis: (1) The process of normalization and removing features for meta-analysis; (2) The step of classification analysis to predict six diseases in integrated metagenome data across the six diseases. (**B**) Number of features at five taxonomy levels. “Total” represents the total number of features before preprocessing of data. “Filtering” represents the number of features after steps for removing features in preprocessing of data.

Metagenome data from 1,079 individuals were collected for the healthy (control samples) and patients with one of six diseases including MS, JIA, ME/CFS, AIDS, Stroke and CRC (Table 1). The study for HIV produced the highest number of average reads (89.9 M) while the study for Stroke had the lowest (4.9 M). Out of all individuals, six individuals with less than 7067.68 reads (<5% of the average) were removed. Thus, the total of 1,073 individuals-696 patients and 377 healthy samples-was used for further analysis. The abundance of microorganisms at the phylum, class, order, family, and genus levels for 1,073 samples were normalized to correct for variations arising from use of different studies (Fig. 1A). After Trimmed Mean of M values (TMM) normalization for the abundance of microorganisms, we compared the abundance of healthy samples from six studies. For the reason to minimize the study-dependent differences, we removed the microorganisms that are differentially abundant between studies (false discovery rate (FDR) < 0.05). Average of 16% of bacteria (5, 21, 42, 74 and 199 at the phylum, class, order, family, and genus levels, respectively) remained (Fig. 1). To further normalize the microbiome abundance of samples from different studies, quantile normalization was performed using the healthy samples as the baseline. The normalized abundance of microorganisms for 696 samples obtained in this preprocessing step was considered as features in the subsequent classification analysis.

**Table 1:** Summary of collected metagenome studies.

SRA_study	Disease	Body site	# of case samples	# of control samples	Average reads per sample (std)
ERP010458	Stroke	Gut	141	92	4.9 M(0.4 M)
ERP013262	JIA	Gut	29	29	9.2 M(2 M)
ERP014628	ME/CFS	Gut	49	39	52.5 M(17.1 M)
SRP068240	HIV1	Gut	191	33	89.9 M(69.9 M)
SRP073172	CRC	Gut	263	141	14.2 M(10.3 M)
SRP075039	MS	Gut	29	44	31.2 M(5.5 M)

### Classification performance at five taxonomy levels

**Figure 2:**
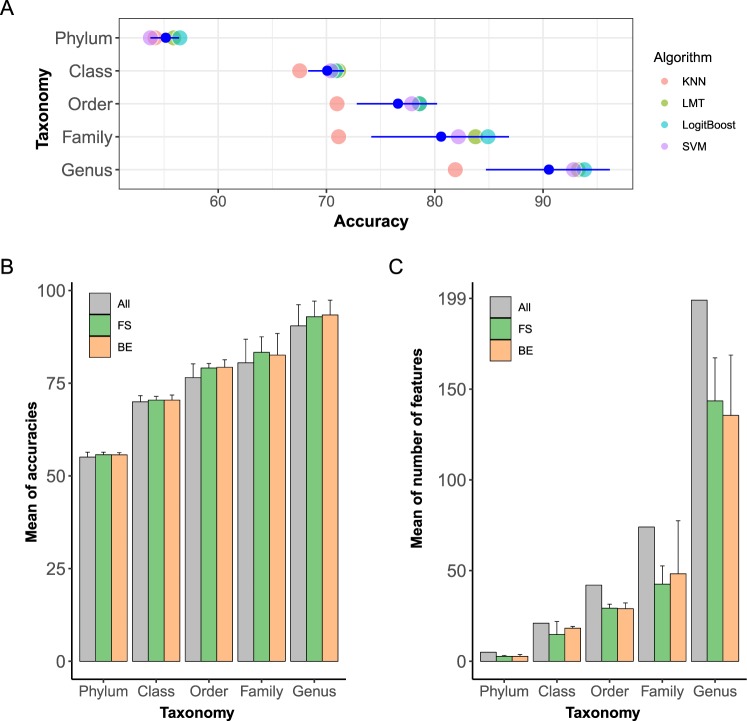
Classification performance by taxonomy levels and feature selection methods. (**A**) Accuracies by taxonomy levels. Individual dots symbolize the accuracy of four classifiers. Blue dots with error bar represents the mean of the accuracies in each taxonomy. (**B**) Mean of Accuracies in four classifiers by taxonomy levels and feature selection method. The color of bars shows the feature selection method. “All” indicates that all features without feature selection are used for classifications. “FS” and “BE” indicates the features subset from FS and BE respectively. Error bar represents the standard error of accuracies at each taxonomy level and feature selection method. (**C**) Mean of number of features in four classifiers by taxonomy levels and feature selection method.

To elucidate the effect of different taxonomy levels on the classification, we assessed the performance of the classification using different sets of features such as the abundance of microorganisms at the phylum, class, order, family, and genus levels. The average of accuracies of four classifiers including KNN, LMT, LogitBoost and SVM was improved as we used the lower taxonomy levels as features (Fig. 2A). The average of accuracies at the phylum, class, order, family and genus levels were 55, 69.9, 76.5, 80.4 and 90.4% respectively. The accuracy at the genus level was 35.4% higher than that at the phylum level. On the other hand, the difference of accuracies between classifiers with highest accuracy (LogitBoost) and lowest accuracy (KNN) was 11.92%. Thus, we found that the effect of taxonomy levels on the classifier performance was greater than that of using different classifiers.

We assumed that some of the microorganisms used in the above classification might not be associated with the diseases because only a few microorganisms were found to be closely related to human health or disease^20^. Hence, we performed feature selection to find features that can classify diseases more accurately. For feature selection, we used forward selection (FS) and backward elimination (BE) in four classifiers with microbial abundance at five taxonomy levels. Feature selection enhanced accuracies by 2.6%, 2.4% and 2.7% at the order, family and genus levels, respectively, while its effects were not as remarkable in phylum and class levels (0.6% and 0.4% enhanced) (Fig. 2B). The highest accuracy improvement of 2.7% due to feature selection was observed when using features of abundance at the genus level. By feature selection, 5, 21, 42, 74, and 199 number of features were reduced to 2.75, 16.5, 29.1, 45.3, and 139.5 on average in phylum, class, order, family and genus levels, respectively (Fig. 2C). The highest number of features was removed at the genus level. Considering the increase of accuracies and number of reduced features, feature selection was more effectively performed at the genus level.

### Comparison of classification performance at the genus level

**Figure 3:**
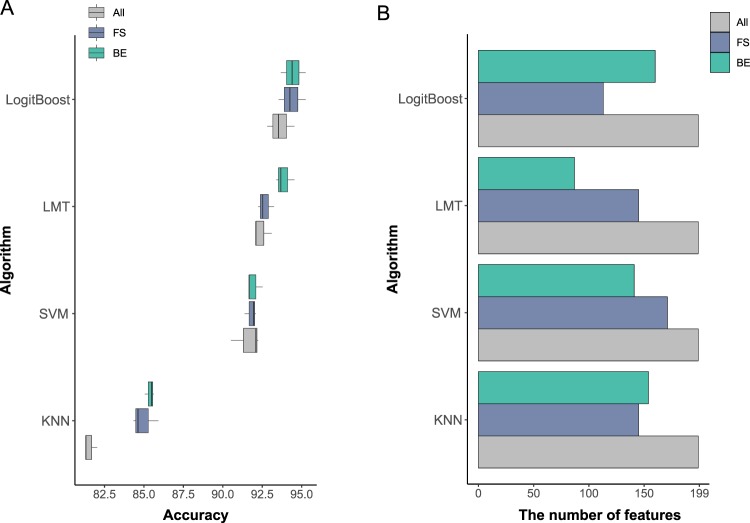
Classification performance by four classifiers at the genus level. (**A**) Accuracies of four classifiers with three feature selection strategies (without feature selection, FS and BE). Evaluation of performance of each model involving different feature selection strategies was conducted three times. (**B**) The number of features by four classifiers with three feature selection strategies.

We compared classifiers and feature selection methods based on the performance at the genus level which showed the highest performance among five taxonomy levels. The classification was conducted using 10-fold cross-validation (CV), and accuracies were averaged over three runs of 10-fold CV. Four classifiers affected the performance of classification (Fig. 3A). The average accuracy was the highest in LogitBoost (93.6%) followed by LMT (92.4%), SVM (91.6%), and KNN (81.5%). The difference of accuracies between classifiers with the highest accuracy (LogitBoost) and that with the lowest (KNN) was 12%. In Fig. 2A, the difference in performance between LogitBoost and KNN increases as the taxonomy level gets lower. Regarding this aspect, the large difference (12%) between LogitBoost and KNN might come from the highest feature number at the genus level.

When we use the optimal feature sets from FS and BE, the average accuracies of the four classifiers were increased from 90.4% to 92.9% and 93.3% (FS and BE). Especially, the accuracies from KNN algorithms showed a remarkable increase from 81.8% to 86.7% and 87.5% when FS and BE were used. In all four classifiers, BE enhanced higher accuracies than FS by 0.09%, 1.19%, 0.09% and 0.43% in LogitBoost, LMT, SVM, and KNN, respectively. In LMT classifier, BE achieved the most effectively enhanced accuracies. The average number of features was reduced from 199 to 143.5 and 135.5 (FS and BE, respectively) across four classifiers (Fig. 3B). Even though BE decreased the number of features much more compared to FS on average, the reduced number of features did not follow this trend in all classifiers. FS effectively reduced the number of features in LogitBoost algorithms, while BE did in LMT algorithm. In summary, performing feature selection enabled us to obtain the subset of features which enhanced the overall performance of the classification in all classifiers. More importantly, higher accuracy was achieved when a lower number of features were used.

### Accuracy, false positive and false negative error rate per six diseases

We examined the classification performance by calculating the accuracy of false positive rate (FPR) and false negative rate (FNR), which is a calculation method used to classify into two classes^21^. Additionally, we investigated the performance of classification per diseases by obtaining feature set from BE with the highest performance. In LogitBoost algorithm, which had the highest performance among classifiers, average accuracy by disease was 98.1%, which is higher than overall accuracy of BE (93.6%) (Table 2). This increase of accuracy was caused by a higher number of true negatives because we applied calculation for evaluating a binomial classification for each disease. For the same reason, the mean of FPR (1.26%) was lower than that of FNR (13.86%). Since FPR divides true positive by sum of a true negative and true positive which makes it inversely proportional to true negative, in our case, as the number of true negative jumps to a greater number, a lower value of FPR was observed. Out of six diseases, CRC showed the highest FPR (3.7%) of all the diseases, which implies the classification of 3.7% of patients with non-CRC diseases as CRC. The lowest accuracy in CRC (96.84%) among six diseases was caused by a highest FPR. As FNR of the diseases showed high variance between diseases, CRC, HIV1, and Stroke (2.28, 0.36, 3.78%) were less than 5% of FNR, whereas JIA, ME/CFS, and MS (16.09, 28.47, 32.18%) were more than 10% of FNR. Diseases with high FNR including JIA, ME/CFS, and MS showed higher occurrences of misclassification into other diseases. In contingency tables, we observed that diseases with a high FNR are highly likely to be classified as CRC which had the highest FNR of all diseases.

**Table 2:** Evaluation of performance per class in feature subset of BE in four algorithms.

	CRC	HIV1	JIA	ME/CFS	MS	Stroke	Average
**Accuracy**							
LogitBoost	**96.84 ± 0.43**	99.71 ± 0.14	98.52 ± 0.22	96.93 ± 0.46	98.28 ± 0.29	98.32 ± 0.46	98.1 ± 0.33
LMT	**95.93 ± 0.3**	98.66 ± 0.22	98.95 ± 0.22	96.26 ± 0.57	98.18 ± 0.44	98.8 ± 0.22	97.8 ± 0.33
SVM	**95.59 ± 0.5**	98.85 ± 0.25	98.28 ± 0.38	96.46 ± 0.08	98.08 ± 0.22	98.75 ± 0.22	97.67 ± 0.28
KNN	**90.28 ± 0.3**	97.27 ± 0.43	97.27 ± 0	94.73 ± 0.36	96.41 ± 0.14	96.55 ± 0.5	95.42 ± 0.29
**FPR**							
	**CRC**	HIV1	JIA	ME/CFS	MS	Stroke	Average
LogitBoost	**3.7 ± 0.83**	0.26 ± 0.11	0.85 ± 0.09	1.18 ± 0.24	0.4 ± 0.09	1.14 ± 0.28	1.26 ± 0.27
LMT	**3.93 ± 0.4**	0.85 ± 0.3	0.6 ± 0	1.7 ± 0.41	0.7 ± 0.43	0.9 ± 0.18	1.45 ± 0.29
SVM	**4.77 ± 0.48**	0.59 ± 0.2	0.8 ± 0.09	1.59 ± 0.09	0.9 ± 0.15	0.6 ± 0.1	1.54 ± 0.19
KNN	**12.93 ± 0.23**	1.83 ± 0.49	1.35 ± 0.15	0.87 ± 0.32	0.4 ± 0.17	2.34 ± 0.18	3.29 ± 0.26
**FNR**							
	CRC	HIV1	**JIA**	**ME/CFS**	**MS**	Stroke	Average
LogitBoost	2.28 ± 0.38	0.36 ± 0.31	**16.09 ± 3.98**	**28.47 ± 9.62**	**32.18 ± 7.18**	3.78 ± 1.48	13.86 ± 3.82
LMT	4.31 ± 0.22	2.69 ± 0	**11.49 ± 5.27**	**31.25 ± 3.61**	**27.59 ± 3.45**	2.36 ± 1.64	13.28 ± 2.37
SVM	3.8 ± 0.66	2.69 ± 1.08	**22.99 ± 7.18**	**29.86 ± 1.2**	**25.29 ± 1.99**	3.78 ± 0.82	14.74 ± 2.16
KNN	4.44 ± 0.44	5.2 ± 0.31	**34.48 ± 3.45**	**64.58 ± 2.08**	**77.01 ± 5.27**	7.8 ± 2.56	32.25 ± 2.35

The model was validated by 10-fold cross-validation and repeated three times. Values represent the mean of accuracy ± variance.

The diseases with high FPR and FNR in other algorithms were the same as that in LogitBoost algorithm. CRC had the highest FPR and the lowest accuracy among diseases in other classifiers. JIA, ME/CFS, and MS had higher FNR compared to other diseases in other classifiers. In KNN algorithm, CRC showed the highest FPR of 12.93%, while other classes showed FPR lower than 3%. Also, FNR of JIA, ME/CFS and MS (34.48, 64.58 and 77.01%) were higher than that of other classes with FNR below 8%. However, classes with higher FPR (or FNR) in KNN showed higher FPR(or FNR) compared to that in LogitBoost. FPR of CRC in KNN (12.93%) was three times higher than that in LogitBoost (3.7%). FNR of JIA, ME/CFS and MS (58.69%; mean of three classes) in KNN was twice as much as that in LogitBoost (25.58%; mean of three classes).

### Identification of the disease-related microbial features

Through feature selections, we detected feature subsets that distinguish six diseases with the highest performance per classifier. Selected features can be used for microbial marker as they may be a shred of evidence of a close relatedness with the six diseases^22^. Thus, we predicted that our selected features could also be applied as biomarkers for the six diseases. Among the potential biomarkers, we examined commonly selected genus in eight selected feature subsets at the genus level from the multiplication of four classifiers and two feature selection methods. The number of common selected features in FS and BE were 94, 66, 120 and 116 in LogitBoost, LMT, SVM, and KNN algorithm, respectively (Fig. S1). Among them, 17 genera were commonly identified in all four classifiers (Table 3). To elucidate further on the importance of these genera in classification, we looked closely into the rank of individual genus. The rank of the genus to be added or dropped during the feature selection procedure could be of interest as the features with greater performance tends to be added earlier or dropped later during feature selection. Therefore, we considered the rank of genus in the selection. Among 17 genera, only PSBM3 was selected in order of no more than five, which is less than 5% of 199 genera (10 number of genera). PSBM3 belongs to a bacterial family called Erysipelotrichaceae, which is associated with immune system^23^. Erysipelotrichaceae was coated by IgA and their abundance had a positive correlation with tumor necrosis factor alpha levels^24,25^. Specifically, PSBM3 is associated with invariant natural killer T, which had a crucial role in pathogenesis of inflammatory diseases^26^.

**Table 3:** Robust genera subset from two feature selection methods in four classifiers.

	Logit Boost/FS	LogitBoost/BE	LMT/FS	LMT/BE	SVM/FS	SVM/BE	KNN/FS	KNN/BE	Mean of order
PSBM3	3	2	5	3	3	2	3	3	3
Candidatus Azobacteroides	6	10	7	8	10	122	5	60	28.5
Cetobacterium	10	19	6	25	19	31	17	154	35.125
Ralstonia	46	17	93	14	27	16	45	24	35.25
Proteus	32	3	126	15	6	27	9	78	37
Flavobacterium	33	7	98	51	44	17	49	7	38.25
Moryella	8	105	1	77	7	1	103	65	45.875
Citrobacter	11	89	20	5	88	7	135	13	46
Anaerofustis	23	6	35	73	66	26	129	36	49.25
Dickeya	18	26	27	10	171	11	28	111	50.25
Owenweeksia	52	16	95	6	8	131	68	58	54.25
Salmonella	22	69	99	61	49	59	125	77	70.125
Pediococcus	99	93	46	82	67	45	145	19	74.5
Variovorax	80	127	54	79	133	79	58	57	83.375
Leuconostoc	83	112	96	63	63	91	94	88	86.25
Marvinbryantia	106	156	118	43	80	113	78	89	97.875
Novosphingobium	51	151	121	48	90	82	116	151	101.25

We present 17 genera selected in combination of four classifiers and two feature selection method. Column represent “Classifier/feature selection method”. The figures in the table show the order of genera in selection steps. The lower number (figure) indicates the more importance for genera in terms of performance.

## Discussion

We compared the performance of classification for six diseases in terms of three factors: 1) taxonomy level, 2) classifier and 3) feature selection method. Among the three factors, altering taxonomy levels influenced the classification performance the most. Moreover, we found that the performance improved as we used lower taxonomy level as features, which is consistent with a previous finding^27^. Microorganisms at lower taxonomy levels have been used to investigate their impact on the host because they help to estimate the function more specifically^28^. This suggests the necessity of using the technology of assigning microorganisms with high resolution in the classification of various diseases. In addition to the taxonomy level, we also evaluated the classification performance of four classifiers. Among the four classifiers, LogitBoost showed the highest performance. LogitBoost algorithm is a boosting model which process interactions effectively and robust to outliers, missing data, and many correlated as well as less important variables^29–32^. This might have a positive influence on enhancing the performance of the classification of multiple diseases. On the other hand, KNN showed the lowest performance. KNN algorithm is reasonably well solved for a smaller number of features^17^. The performance of KNN algorithm was especially lower at the genus level compared to the other classifiers.

We constructed feature subsets using FS and BE. FS and BE achieve improved accuracy because they find the optimal feature sets by interacting with classifiers^33^. On the other hand, FS and BE require expensive computation times with a large number of features. This might rarely cause their application in the gut microbiome data. In this study, we showed that the selected microorganisms with FS and BE could boost the performance, especially, the feature subsets selected by BE had higher performance than that by FS. Since BE starts with the full set of features, it is easier to capture the interactive features, such that this advantage of BE can take into account the complex network of microbe-microbe interactions. Microbes interact with each other by forming microbial guilds where they provide the substrate to each other, and even some anaerobic bacteria in the gut were demonstrated to perform metabolic cross-feeding^19^. Therefore, a group of microorganisms is more related to human health than individual ones, which is why a higher performance of BE was observed.

While performing the feature selection, we proposed the feature subsets that are potentially related to six different diseases. However, the feature subsets selected in this study may not contain all the microorganisms associated with the six diseases due to the data preprocessing. We preprocessed the data with various measures such as employing strict criteria when collecting the data from various studies and performing TMM and quantile normalization to minimize the variations between the studies. In addition, the samples were composed of a variety of nationalities which influence dietary habits, thereby affecting the composition of the gut microbiome. Some features, which might be affected by variation among samples, were deleted to reduce heterogeneity across different studies, which might cause by the effect of nationality. Thus, a few features significantly related to the six diseases may have been removed from this process. Despite the limitation of data preprocessing from different studies, we detected microorganisms associated with the six diseases.

Association with gut microbiome and health suggested the potential roles of gut microorganisms in precision medicine approach^34^. Disease-related microorganisms can be used as microbial markers to detect diseases using well-known methods including metagenomics, phylogenetic microarrays, DNA fingerprinting techniques, and qPCR^26^. Most of the previous disease studies on metagenome data focused on identification of biomarkers by comparing two groups of samples (case-control study)^35^. However, focusing on one disease may not be able to detect biomarker bacteria that is specific to that disease. This is because the same microorganisms can be differentially abundant in several diseases since the immune system of the host is influenced by the certain gut microbiome community that can be vulnerable to various diseases^8,36^. On the other hand, the selected features in this study are expected to have disease specific profiling of microbial communities, which can be used for biomarkers to distinguish various diseases simultaneously. For example, PSBM3 (belongs to Family Erysipelotrichaceae) was an important feature in eight feature subsets. In the previous study, family Erysipelotrichaceae was studied to be associated with host diseases such as inflammatory bowel disease and HIV, as well as with the immune system^23–25^. This implies that the abundance of family Erysipelotrichaceae (or genus PSBM3) is an important clue to detecting multiple diseases.

As a result of the classification per diseases investigation, we found that JIA, ME/CFS and MS are classified into CRC. According to previous studies, CRC is related to fatigue symptom, which is a similar symptom with ME/CFS^37^. The fatigue by CRC can be affected by sarcopenia, characterized by muscle loss, which demonstrates the relationship between ME/CFS and CRC^38^. Moreover, there is a possible relationship between cancer risk and MS, which can cause diagnostic neglect^39^. Though, the association between CRC and JIA has not been identified.

In summary, we presented the classification of six diseases using a machine learning algorithm and gut microbiome data. By evaluating performance in various perspectives, we showed the effect of bacterial abundance of different taxonomy levels and various classifier on performance of classification. Furthermore, we suggested the optimal genus subsets that can be potentially used as microbial markers to distinguish multiple diseases through feature selection, which confers the potential use for multi-diseases classification in the diagnosis of diseases.

## Materials and Methods

### Collection of the gut microbiome data related to six diseases

For disease prediction based on the metagenome data sets of gut microbial communities, large numbers of metagenome samples were collected from the European Bioinformatics Institute (EBI) database (https://www.ebi.ac.uk/metagenomics/). To minimize the biases caused by different experimental protocols, data were collected with several criteria: (1) 16 S rRNA based metagenome data through the stool sampling, which is widely used approach at present, (2) sequencing platforms including 454 and Illumina’s, (3) using first measurement in case of longitudinal data to ensure independence assumption and (4) EBI pipeline v2.0 or v3.0 (https://www.ebi.ac.uk/metagenomics/pipelines/3.0) for identifying and quantifying the OTUs. In EBI pipeline, several tools used are as following: (1) Trimmomatic (v0.32)^40^ for quality check and trimming of low quality reads; (2) SeqPrep (v1.1)^41^ to merge paired-end reads to generate overlapped read; (3) rRNASelector (v1.0.1)^42^ to filter out of non-ribosomal RNA; (4) QIIME(v1.9.0)^43^ for OTU identification and quantification. From this pipeline, gut microbial communities data was generated at various taxonomic levels such as phylum, class, order, family, and genus based on the Greengenes 16 S rRNA database^44^.

### Preprocessing of the metagenomic data derived from different studies

Samples with less than 5% of the average number of reads were removed. The abundance of microorganisms at five taxonomy levels including phylum, class, order, family, and genus levels was used as features. We performed a TMM normalization for the abundance of features using edgeR^45^. To reduce heterogeneity across different studies, the features showing differential abundance of healthy samples between six studies were removed. We performed a log- likelihood ratio test by considering the abundance of features as negative binomial distribution^46^. In the statistical test, FDR approach was used to adjust multiple testing error^47^ and 5% significance level was used for a significant result.

We further normalized the abundance with quantile normalization to produce a similar distribution of samples^48^. For quantile normalization, two types of baselines can be considered to calculate normalized values: (1) global mean vector derived from each quantile of features and (2) specific baseline vector. As we assumed that distribution of all control samples are similar, the second approach was employed using only the healthy samples to create the baseline^49^.

### Classifiers to distinguish various diseases using the gut microbial data

In this study, four classifiers which have previously shown high multi-group classification performance were employed including KNN, LogitBoost, LMT and SVMs with sequential minimal optimization (SMO)^50,51^. The KNN implies a classifier capable of multi-groups classification. The LogitBoost is a developed boosting algorithm that can handle multiclass problems by considering multiclass logistic loss^52^. The LogitBoost has been applied to predict protein structural classes^53^ and places of origin for pigs with high performance^54^. The LMT is based on a regression tree that has logistic models on the leaves^51^. In predictions related to medical application including prediction of response to antiretroviral combination therapy or autism spectrum disorder, LMT showed an advantage over the other methods^55,56^. The SMO has been shown to be an effective method for SVM on classification tasks without a quadratic programming solver. The KNN and SVM classifiers are the most widely used methods and they have been applied successfully in numerous studies^17,54^.

We performed classification analysis with the four classifiers, implemented in the RWeka package of the R software^57^ with the command line of *“IBk(class~.,data* = *InputData, control* = *Weka_control(K* = *Selected Parameter), na.action* = *NULL)”, “LogitBoost(class~.,data* = *InputData, control* = *Weka_control(I* = *Selected Parameter), na.action* = *NULL)”, “LMT(class~.,data* = *InputData, na.action* = *NULL)”, and SMO(class~.,data* = *InputData, control* = *Weka_control(K* = *list(kernel, G* = *Selected Parameter), C* = *Selected Parameter), na.action* = *NULL)* for KNN, LogitBoost, LMT, and SVM. To assess the performance of classification, 10-fold cross-validation was used.

To select a parameter for the classifier, we used a greedy method that explores all parameter and used the parameter with the best performance. In KNN, parameter K was chosen in {3, 5, 7, 9, 11, 13, 15} (Table S1). In LogitBoost, the parameter I was selected in the range from 1 to 40 (Table S2). In SVM (for RBF kernel), the parameter G and parameter C were regulated in {1e-4, 1e-3…, 10} and {0.1, 1, …, 1000} respectively (Table S3). The parameters with the highest accuracy were chosen for each taxonomy level (Table S4). For the parameters with same accuracy, the one with lower value was selected.

### Feature selection using wrapper method

We searched for a feature subset that enhances performance of classification through a wrapper feature-selection approach^58^ including FS and BE^54^. In FS, starting from the single feature with the highest accuracy, we added the feature that improves the performance the most. We continued to add features one-by-one until no more feature is left to be added. In BE, starting with all features we subtracted features one-by-one to give the highest accuracy. With the feature selection process, we obtained the feature subset showing the highest accuracy.

## Supplementary information

Supplementary Information

## Data Availability

Raw sequencing data and patient metadata are available at the NCBI Sequence Read Archive (SRP073172, SRP068240, ERP013262, ERP014628, SRP075039 and ERP10458).
