# Supplementary material for: Establishment and evaluation of prediction model for multiple disease classification based on gut microbial data
Source: Sci Rep. 2019 Jul 15;9:10189. doi: 10.1038/s41598-019-46249-x (PMC6629854; doi:10.1038/s41598-019-46249-x)
Supplement: Supplementary file 1 — Supplementary Information [file 41598_2019_46249_MOESM1_ESM.docx]

**Supplementary Information**

**Establishment and evaluation of prediction model for multiple disease classification based on gut microbial data**

**Sohyun Bang****^1,2*^, DongAhn Yoo****^1^, Soo-Jin Kim^3^, Soyun Jhang^1,2^, Seoae Cho^2^, Heebal Kim^1,2,3§^**

^1^Interdisciplinary Program in Bioinformatics, Seoul National University, Seoul 151-742, Republic of Korea.

^2^C&K genomics, Seoul National University Research Park, Seoul 151-919, Republic of Korea.

^3^Department of Agricultural Biotechnology and Research Institute of Agriculture and Life Sciences, Seoul National University, Seoul, Republic of Korea.

^*^ First author

^§^ Corresponding author

Heebal Kim: Tel: +82-2-880-4803, Fax: +82-2-883-8812; E-mail: heebal@snu.ac.kr

Figure S1. The number of intersect of selected features from FS and BE


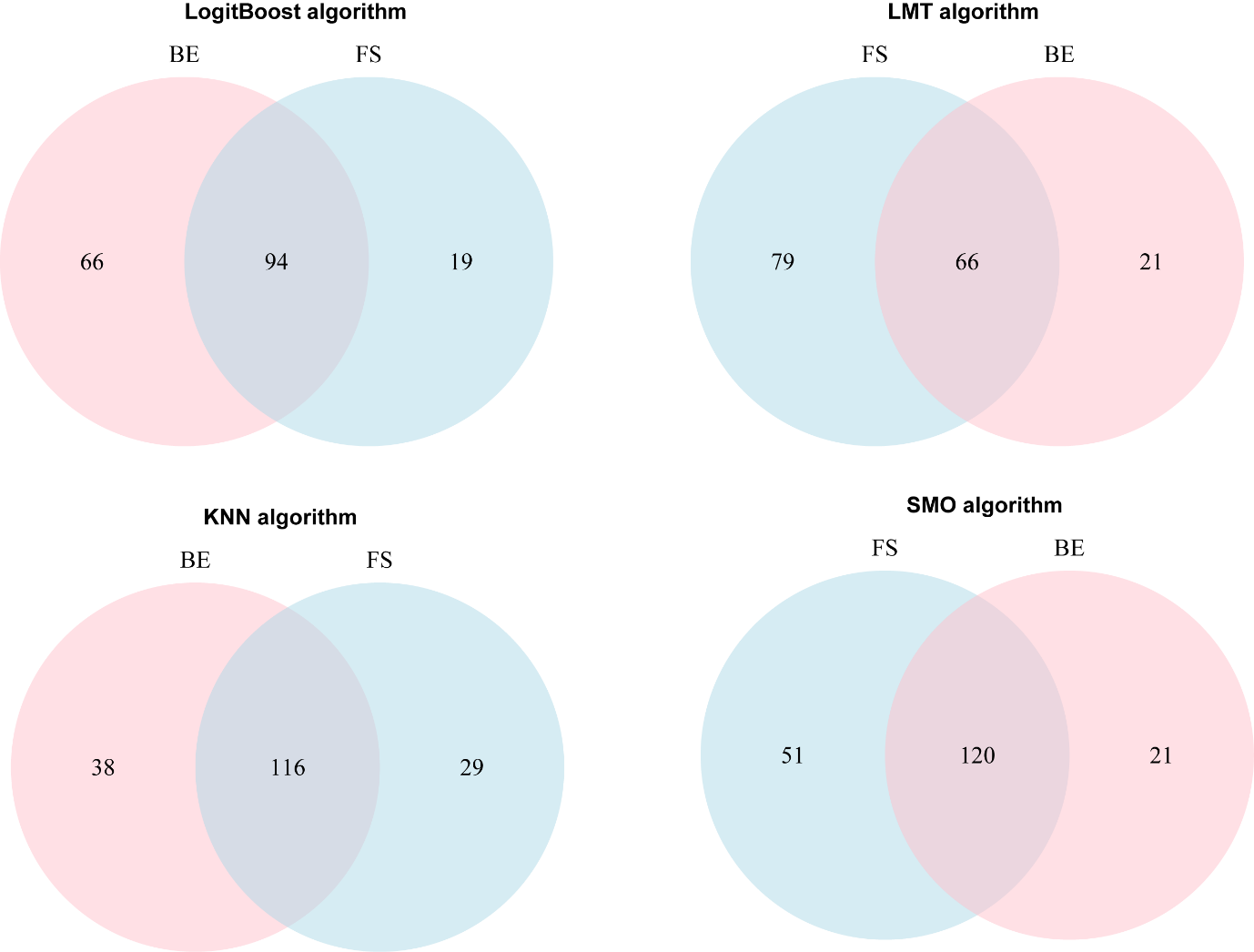


Table S1. Parameters for KNN algorithm. The highest accuracy was shown in bold for each taxonomy level.

| Parameter (K) | Accuracy | | | | |
| --- | --- | --- | --- | --- | --- |
|  | Phylum | Class | Order | Family | Genus |
| 3 | **53.88** | **66.95** | **71.12** | 70.40 | 81.75 |
| 5 | 52.73 | 65.80 | 70.98 | 68.82 | 81.75 |
| 7 | 53.30 | 64.22 | 69.54 | 69.11 | **82.18** |
| 9 | 53.45 | 63.07 | 69.54 | 70.11 | 81.47 |
| 11 | 53.16 | 62.21 | 69.25 | **71.12** | 82.18 |
| 13 | 53.30 | 61.93 | 69.11 | 70.55 | 81.61 |
| 15 | 53.88 | 60.20 | 67.10 | 70.40 | 80.75 |

Table S2. Parameters of LogitBoost algorithm. The highest accuracy was shown in bold for each taxonomy level.

| Parameter (I) | Accuracy | | | | |
| --- | --- | --- | --- | --- | --- |
|  | Phylum | Class | Order | Family | Genus |
| 1 | 52.16 | 60.34 | 65.52 | 71.70 | 77.73 |
| 2 | 53.30 | 64.37 | 69.40 | 71.70 | 81.18 |
| 3 | 55.03 | 67.53 | 71.12 | 77.59 | 84.63 |
| 4 | **57.18** | 67.53 | 71.55 | 78.45 | 85.92 |
| 5 | 55.75 | 68.53 | 75.14 | 79.89 | 88.07 |
| 6 | 54.60 | 70.11 | 74.86 | 80.75 | 91.09 |
| 7 | 55.17 | 70.40 | 76.29 | 81.47 | 90.66 |
| 8 | 55.03 | 69.97 | 76.15 | 82.04 | 91.24 |
| 9 | 54.60 | 70.26 | 77.59 | 81.61 | 91.95 |
| 10 | 55.60 | 70.69 | 76.01 | 82.61 | 91.95 |
| 11 | 55.03 | 70.98 | 77.01 | 82.90 | 92.82 |
| 12 | 54.60 | 70.98 | 77.87 | 83.05 | 92.67 |
| 13 | 55.32 | 70.69 | 76.72 | 82.18 | 92.67 |
| 14 | 54.60 | 70.83 | 77.73 | 83.19 | 93.39 |
| 15 | 54.45 | 70.98 | 77.59 | 82.47 | 93.82 |
| 16 | 54.74 | 70.55 | 78.02 | 83.48 | 92.82 |
| 17 | 53.88 | 70.69 | 77.44 | 84.05 | 94.54 |
| 18 | 54.89 | 70.83 | 78.02 | 83.91 | 92.67 |
| 19 | 54.89 | 70.40 | 76.87 | 83.91 | 93.97 |
| 20 | 54.89 | 71.12 | 78.45 | 85.06 | 93.97 |
| 21 | 54.60 | 70.69 | 78.45 | 84.77 | 94.54 |
| 22 | 55.17 | 71.12 | 77.44 | 84.77 | 94.68 |
| 23 | 54.60 | **71.55** | 77.87 | 84.63 | 94.54 |
| 24 | 55.03 | 70.40 | **79.31** | 84.20 | 94.40 |
| 25 | 54.89 | 70.83 | 79.02 | 84.48 | 93.39 |
| 26 | 54.60 | 70.69 | 79.31 | 84.34 | 93.53 |
| 27 | 55.32 | 71.12 | 77.16 | 83.62 | 94.25 |
| 28 | 54.60 | 70.83 | 77.87 | 84.77 | 93.97 |
| 29 | 54.45 | 70.98 | 77.16 | 84.20 | 94.68 |
| 30 | 55.75 | 69.83 | 77.87 | **85.49** | **95.40** |
| 31 | 54.89 | 71.12 | 77.73 | 84.48 | 94.40 |
| 32 | 54.89 | 70.26 | 78.02 | 84.48 | 95.11 |
| 33 | 54.45 | 70.55 | 78.16 | 84.63 | 93.97 |
| 34 | 55.46 | 70.83 | 78.45 | 83.33 | 93.82 |
| 35 | 54.74 | 70.98 | 77.87 | 84.05 | 94.68 |
| 36 | 55.03 | 70.55 | 78.45 | 84.63 | 94.11 |
| 37 | 55.32 | 70.83 | 78.59 | 82.90 | 94.97 |
| 38 | 54.45 | 70.40 | 79.02 | 84.77 | 93.97 |
| 39 | 54.89 | 70.69 | 79.02 | 84.63 | 94.11 |
| 40 | 54.89 | 70.98 | 77.30 | 83.48 | 94.83 |

Table S3. Parameter of RBF Kernel in SVM algorithm. The columns represent parameter G, while the rows represent parameter C. The highest accuracy was shown in bold for each taxonomy level.

|  | **Phylum** | | | | | |
| --- | --- | --- | --- | --- | --- | --- |
|  | 1e.04 | 0.001 | 0.01 | 0.1 | 1 | 10 |
| 0.1 | 37.79 | 37.79 | 37.79 | 52.59 | 54.31 | 55.03 |
| 1 | 37.79 | 37.79 | 52.44 | 54.89 | 54.17 | **55.89** |
| 10 | 37.79 | 50.86 | 54.89 | 54.60 | 54.74 | 54.45 |
| 100 | 52.44 | 54.89 | 55.60 | 55.32 | 55.03 | 54.74 |
| 150 | 53.45 | 54.89 | 55.17 | 54.45 | 54.17 | 55.03 |
| 200 | 53.59 | 54.89 | 55.03 | 54.74 | 55.03 | 54.89 |
| 300 | 53.74 | 54.89 | 55.32 | 54.89 | 55.32 | 54.89 |
| 400 | 54.31 | 54.89 | 54.31 | 54.45 | 54.17 | 54.45 |
| 1000 | 54.89 | 55.03 | 55.32 | 54.60 | 55.17 | 54.31 |
|  | **Class** | | | | | |
|  | 1e.04 | 0.001 | 0.01 | 0.1 | 1 | 10 |
| 0.1 | 37.79 | 37.79 | 37.79 | 54.60 | 64.66 | 48.85 |
| 1 | 37.79 | 37.79 | 56.18 | 63.79 | 68.68 | 68.97 |
| 10 | 37.79 | 56.18 | 61.49 | 69.11 | 69.25 | 68.68 |
| 100 | 56.32 | 61.35 | 68.97 | 70.40 | 69.40 | 68.68 |
| 150 | 59.63 | 68.25 | 69.11 | 70.11 | 70.26 | 68.53 |
| 200 | 60.34 | 68.68 | 69.97 | 70.40 | 69.97 | 68.82 |
| 300 | 60.34 | 68.25 | 70.11 | 69.97 | 69.83 | 68.97 |
| 400 | 60.78 | 68.82 | 70.26 | 69.68 | 69.40 | 68.82 |
| 1000 | 62.50 | 68.68 | **70.55** | 69.54 | 70.11 | 68.39 |
|  | **Order** | | | | | |
|  | 1e.04 | 0.00 | 0.01 | 0.10 | 1.00 | 10.00 |
| 0.1 | 37.79 | 37.79 | 37.79 | 51.58 | 69.40 | 41.38 |
| 1 | 37.79 | 37.79 | 60.20 | 74.57 | 78.02 | 59.91 |
| 10 | 37.79 | 61.64 | 75.43 | 77.87 | 75.86 | 61.93 |
| 100 | 59.63 | 74.43 | **79.31** | 76.87 | 74.86 | 61.78 |
| 150 | 68.97 | 76.29 | 77.87 | 78.59 | 76.15 | 61.49 |
| 200 | 69.40 | 76.29 | 78.02 | 78.45 | 76.01 | 63.22 |
| 300 | 71.70 | 77.73 | 77.16 | 78.02 | 76.58 | 61.49 |
| 400 | 72.41 | 78.16 | 77.73 | 78.02 | 75.14 | 62.36 |
| 1000 | 75.29 | 78.30 | 78.59 | 76.87 | 75.86 | 61.78 |
|  | **Family** | | | | | |
|  | 1e.04 | 0.00 | 0.01 | 0.10 | 1.00 | 10.00 |
| 0.1 | 37.79 | 37.79 | 37.79 | 49.43 | 51.58 | 37.79 |
| 1 | 37.79 | 37.79 | 59.91 | 77.73 | 78.30 | 38.22 |
| 10 | 37.79 | 62.07 | 78.88 | 81.90 | 78.30 | 39.22 |
| 100 | 61.49 | 78.59 | 82.61 | 79.45 | 79.17 | 38.94 |
| 150 | 68.68 | 79.17 | 82.04 | 80.17 | 78.30 | 39.08 |
| 200 | 70.98 | 79.17 | 81.90 | 77.59 | 79.89 | 38.79 |
| 300 | 74.14 | 80.60 | **82.76** | 79.74 | 79.17 | 38.79 |
| 400 | 75.57 | 81.03 | 82.61 | 79.45 | 79.02 | 39.22 |
| 1000 | 78.45 | 82.61 | 81.75 | 80.17 | 79.45 | 38.94 |
|  | **Genus** | | | | | |
|  | 1e.04 | 0.00 | 0.01 | 0.10 | 1.00 | 10.00 |
| 0.1 | 37.79 | 37.79 | 37.79 | 62.21 | 39.08 | 37.79 |
| 1 | 37.79 | 37.79 | 69.11 | 86.06 | 79.31 | 37.79 |
| 10 | 37.79 | 69.40 | 1.00 | 91.52 | 81.18 | 37.79 |
| 100 | 69.54 | 88.22 | 92.53 | 91.09 | 81.47 | 37.79 |
| 150 | 77.30 | 90.23 | 91.81 | 92.24 | 81.75 | 37.79 |
| 200 | 79.02 | 91.52 | 91.95 | 92.53 | 82.04 | 37.79 |
| 300 | 80.75 | 91.09 | 92.10 | 91.67 | 81.18 | 37.79 |
| 400 | 82.76 | 91.81 | 93.10 | 91.95 | 80.46 | 37.79 |
| 1000 | 87.21 | 92.53 | **93.25** | 92.39 | 81.32 | 37.79 |

Table S4. Selected parameter in disease classification.

|  | **KNN** | **LogitBoost** | **SMO** | |
| --- | --- | --- | --- | --- |
|  | **K** | **I** | **C** | **G** |
| **Phylum** | 3 | 4 | 1 | 10 |
| **Class** | 3 | 23 | 1000 | 0.01 |
| **Order** | 3 | 24 | 100 | 0.01 |
| **Family** | 11 | 30 | 300 | 0.01 |
| **Genus** | 7 | 30 | 1000 | 0.01 |
